# Repurposing of high-dose *N*-acetylcysteine as anti-inflammatory, antioxidant and neuroprotective  agent  in moderate to severe traumatic brain injury patients: a randomized controlled trial

**DOI:** 10.1007/s10787-025-01706-0

**Published:** 2025-04-10

**Authors:** Alaa Refaat Gouda, Noha A El-Bassiouny, Ahmad Salahuddin, Emad Hamdy Hamouda, Amira B. Kassem

**Affiliations:** 1 Clinical Pharmacy and Pharmacy Practice Department, Faculty of Pharmacy, Damanhour University, Damanhour, 22514 Egypt; 2 Biochemistry Department, Faculty of Pharmacy, Damanhour University, Damanhour, 22514 Egypt; 3 Department of Biochemistry, College of Pharmacy, Al-Ayen Iraqi University, Nasiriyah, Thi-Qar 64001 Iraq; 4 Critical Care Medicine Department, Faculty of Medicine, University of Alexandria, Alexandria, 21517 Egypt

**Keywords:** Traumatic brain injury, *N*-Acetylcysteine, Neuron-specific enolase, Malondialdehyde, S100B, Glasgow Coma Scale

## Abstract

**Introduction:**

Traumatic brain injury (TBI) refers to an impact of the brain within the skull resulting in an altered mental state. The study aim is to determine the effect of a high dose of *N*-acetylcysteine (NAC) on biochemical and inflammatory markers of neuronal damage and clinical outcomes in patients with moderate to severe TBI.

**Methods:**

A randomized open label-controlled trial was conducted on 40 patients with moderate to severe TBI patients presented to the emergency unit within < 24 h since the trauma occurred and randomized into NAC and control groups 20 patients each. Serum samples for evaluation of biomarkers: malondialdehyde (MDA), interleukin-6 (IL-6), neuron-specific enolase (NSE), and S100B were withdrawn at baseline and on day 7. The patients were followed for 7 days and evaluated clinically by the Glasgow Coma Scale (GCS).

**Results:**

There was a significant decrease in NSE and MDA levels on day 7 from baseline in NAC group (*p* < 0.001 and *p* < 0.001). Also, S100B and IL-6 decreased significantly in NAC group on day 7 from baseline (*p* = 0.003 and *p* < 0.001 consequently) compared to control group. Moreover, patients in NAC group showed a significantly shorter length of stay at intensive care unit (ICU) (*p* = 0.038). There was a significant increase in GCS in NAC group on day 7 from baseline (*p* = 0.001).

**Conclusion:**

Adjunctive early use of high-dose NAC significantly reduced inflammatory and oxidative markers and had neuroprotective effect which may be a novel treatment option for moderate to severe TBI patients.

**Trial registration:**

Pactr.org identifier: (PACTR202209548995270) on 14 September 2022.

## Introduction

Traumatic brain injury (TBI) is a crucial cause of mortality and morbidity globally (Syafrita and Fitri 2021; Kalra et al. 2022). The incidence reaches millions of cases worldwide each year and the post-TBI disability rate is of high concern in both young and geriatric populations (Syafrita and Fitri 2021). TBI patients are more likely to suffer from various consequences ranging from short and long-term emotional, behavioral, and physical disabilities to prolonged or even irreversible neurocognitive impairment as changes in cognition, motor function, personality, and neurological diseases such as Alzheimer’s disease (AD) and Parkinson’s disease (PD) (Eakin et al. 2014; Kaur and Sharma 2017; Ghiam et al. 2021). There is currently no approved treatment for TBI that can help patients’ recovery, decrease TBI consequences, or improve long-term clinical outcomes or patients’ quality of life (Ghiam et al. 2021).

The pathophysiology of TBI involves a primary injury that is caused by mechanical forces that compromise the integrity of brain cells and happens at the precise moment of insult (Pearn et al. 2017). The severity of a primary injury might range from asymptomatic to mild to moderate to severe (Pearn et al. 2017). Secondary injury involves several physiological processes, including free radical damage, inflammation, and glutamatergic excitotoxicity (Lenzlinger et al. 2001). The extent of brain damage cannot always be accurately assessed by usual examinations, including assessment of the level of consciousness and imaging examinations, which are frequently carried out once the patient arrives at the hospital (McHugh et al. 2007). This is because the primary injury is followed by a continuous sequence of secondary injuries (McHugh et al. 2007). Therefore, the prognosis of brain injury cannot always be predicted by early imaging. So, the measurement of serum biomarker levels can early predict pathological conditions and outcomes in TBI patients (Syafrita and Fitri 2021).

Interleukin-6 (IL-6), a pro-inflammatory cytokine, has been associated with unfavorable long-term clinical outcomes and increased mortality rates. (Ferreira et al. 2014; Nwachuku et al. 2016). Also, high concentrations of IL-6 were associated with multiple organ failure, sepsis, and poor neurological outcomes (Lustenberger et al. 2016). The serum IL-6 levels identify TBI patients who developed elevated intracranial pressure (ICP) (Hergenroeder et al. 2010).

Malondialdehyde (MDA) is a marker for lipid peroxidation, which increases in individuals with TBI owing to increased oxidative stress (Pietro et al. 2020). Early after a TBI, oxidative stress occurs, resulting in increased lipid peroxidation and reduced enzymatic antioxidant activity, which might affect clinical outcomes in these individuals (Pietro et al. 2020). High levels of MDA have been linked to worse survival and poor outcomes in patients with TBI. MDA was discovered to be a major predictor of TBI recovery (Muballe et al. 2019).

S100 calcium-binding protein B (S100B) and neuron-specific enolase (NSE) are specific neurobiochemical markers of brain injury as they are mainly found in the cytoplasm of neurons and not secreted into the extracellular liquid unless cell destruction occurs (Ann Liebert et al. 2000). The levels of S100B and NSE are closely related to the prognosis of patients with severe TBI (Gao et al. 2021). Increased serum S100B levels constitute a strong predictor of undesirable outcomes, extended Glasgow Coma Scale (GCS), and long-term disability in TBI patients (Da Rocha et al. 2006; Elsayed Abdelfattah et al. 2021).

N-acetylcysteine (NAC), a derivative of the amino acid cysteine, serves as a precursor for the synthesis of glutathione (GSH), a key antioxidant in the body (Chen et al. 2008). NAC acts by upregulating the level of GSH within the brain by scavenging free radicals and functioning as an antioxidant (Chen et al. 2008; Joy et al. 2018). NAC, commonly recognized as an antidote for acetaminophen overdose, is increasingly gaining attention as a potential therapy for both vascular and non-vascular neurological disorders. In numerous animal models, NAC has demonstrated strong neuroprotective benefits, especially in ameliorating the effects of secondary neuronal injury of TBI (Xiong et al. 1999). In rat and mouse models, NAC is effective in treating not just TBI, but also subsequent neurodegenerative disorders associated with TBI. It also decreases tau and beta-amyloid deposition and functions as an anti-inflammatory drug in treating AD through the upregulation of GSH (Joy et al. 2018; Tardiolo et al. 2018).

Few experimental studies have studied the role of NAC in different human populations as antioxidant, anti-inflammatory, and neuroprotective (Amen et al. 2011; Hoffer et al. 2013; Clark et al. 2017; Zhang et al. 2018; Sabetghadam et al. 2020). The first study used a combination of NAC, multivitamins, and weight loss on retired professional football players in an outpatient neuropsychiatric clinic revealed statistically significant increases in microcog assessment of cognitive functioning and brain single-photon emission computed tomography (SPECT) imaging and cognitive and cerebral blood flow improvements (Amen et al. 2011). The first randomized controlled study examined short-term sequelae of mild TBI demonstrated that early administration within 24 h of oral NAC had beneficial outcomes on the severity and resolution of sequelae of mild TBI as dizziness, headache, memory and hearing loss, sleep disturbances, and neurocognitive dysfunction (Hoffer et al. 2013). Another study was performed on children with severe TBI and investigated combination therapy of NAC, as an antioxidant, and probenecid revealing that there were no adverse events attributable to drug treatment (Clark et al. 2017). Also, Early administration of oral NAC by Hoffer et al. regimen (4 g of NAC as loading dose followed by 2 g every 12 h for 4 days then 1.5 g every 12 h for the subsequent 3 days), was associated with improving neurological outcomes of patients with acute ischemic stroke due to its antioxidant and anti-inflammatory effects (Sabetghadam et al. 2020). Other study was performed on community-acquired pneumonia (CAP) patients and investigated the addition of NAC to treatment therapy, reducing MDA suggesting promising antioxidant and anti-inflammatory effects of NAC in pneumonia (Zhang et al. 2018). However, there is no previous interventional study has investigated the potential effect of high-dose NAC in this special population of moderate and severe TBI patients.

The aim of the study was mainly to evaluate the effect of early administration  of high-dose NAC on moderate to severe TBI patients by evaluating neurological improvement through measuring serum levels of two newly promising biomarkers NSE and S100B at baseline and on day 7, in addition to evaluating the antioxidant and anti-inflammatory effect of NAC through measuring serum levels of MDA and IL-6 at baseline and on day 7 and evaluating neurological clinical improvement through GCS score, 28-day mortality, ventilation demand, and intensive care unit (ICU) length of stay compared to control group who received standard care only.

## Design and methods

A randomized open-label prospective controlled trial in moderate to severe TBI patients was conducted from September 2022 to January 2024 at Alexandria Main University Hospital, Alexandria University, Alexandria, Egypt). The study protocol was registered at pactr.org (PACTR202209548995270). The study included two arms, NAC group (20 patients) and control group (20 patients). All patients were assessed for baseline characteristics, vital signs, GCS, Revised Trauma Score (RTS), brain computed tomography (CT) scan, and Rotterdam CT score at baseline. Also, peripheral venous whole blood was obtained at admission as a baseline before NAC administration and on day 7 to measure NSE, S100B, MDA, and IL-6 levels.

### Study population, randomization, and eligibility

Forty patients who met the inclusion/exclusion criteria were randomized by block randomization method to either the NAC group (intervention group; *n* = 20) or the control group (*n* = 20). All patients aged 18–90 years with moderate GCS (9–12) or severe GCS (3–8) TBI and able to tolerate enteral feeding presenting to the critical care resuscitation unit at the Alexandria Main University Hospital within the first 24 h of injury were screened for eligibility. For eligible patients, written informed consent was obtained from their legally authorized representatives before enrollment. The patients were excluded if they were lately presented to the hospital > 24 h or had a pre-existing hepatic failure (Serum alanine aminotransferase (ALT) and serum aspartate aminotransferase (AST) greater than 3 times the upper limit of normal), pre-existing renal failure (blood urea nitrogen (BUN)/ Creatinine 20:1; creatinine > 2 mg/dL) or pregnant and breast-feeding women.

### Intervention

The NAC group received a regimen based on the dose used by Hoffer et al. (Hoffer et al. 2013) orally or by feeding tube: Loading dose of 4 g followed by 2 g every 12 h for 4 days followed by 1.5 g every 12 h for 3 days, in addition to the standard treatment for 7 days. The control group received standard treatment for 7 days. The patients were followed for 7 days and were evaluated clinically by GCS. All patients were followed for hospital stay days and 28-day mortality by contact with patients or their relatives.

Standard care according to the hospital's protocol was provided to the NAC group and control group. Based on the neurologic status and findings on the brain CT, emergency surgical interventions were performed if needed. Standard care includes continuous oxygen supplementation via nasal cannula, face mask, or mechanical ventilation for hypoxemia. Tranexamic acid or blood transfusion to manage bleeding and coagulopathy. Sedative medications, hyperosmolar therapy, hyperventilation, cerebrospinal fluid drainage, and decompressive craniectomy for controlling elevated ICP. Prophylactic antiseizure medication for 7 days after the TBI to reduce the risk of early posttraumatic seizures. Low-molecular-weight heparin (LMWH) or low-dose unfractionated heparin (UH) in combination with mechanical prophylaxis to reduce the risk of deep vein thromboembolism in stable patients. Also, monitoring vital signs and early rehabilitation were considered.

### The outcome measures

Measurement of the serum level of IL-6 and MDA to assess the anti-inflammatory and antioxidant effect of NAC, measurement of NSE and S100B to assess the neuroprotective effect of NAC at baseline and on day 7. Also, evaluating neurological clinical improvement through GCS score at baseline and on day 7, 28-day mortality, ventilation demand, and ICU length of stay compared to control group who received standard care only.

### Analysis of inflammatory, oxidative stress, and neurological biochemical markers

To measure IL-6, MDA, NSE, and S100B levels, peripheral venous whole blood was obtained on admission before NAC administration at baseline and on day 7 after randomization. The serum was separated by centrifugation (8000 rpm for 20 min) in a normal centrifuge. The supernatant was transferred to a microtube and frozen immediately at − 80 °C freezer until analysis. The analysis was performed with commercially available enzyme-linked immunosorbent assay (ELISA) kits purchased from Wuxi Donglin Sci & Tech Development Co., Ltd. Company, china. All collected patients' samples were assayed simultaneously.

### Monitoring of adverse effects and events

All patients were monitored for any adverse events and drug adverse effects during the study period. Clinical follow up and lab investigations as complete blood count (CBC), renal function tests, liver function tests, electrolytes and coagulation profile were monitored.

### Sample size

Based on a study by Mansour et al. (2021), a minimal total sample size of 20 participants (10 in each group), is needed to determine the difference between the two study groups in biomarkers serum levels on day 7, using two-sample *T* test power analysis that detect a difference of 4 ng/mL between both groups that achieves 80% power with a significance level of 5%.

### Statistical analysis

Descriptive statistics were expressed by means and standard deviations. The baseline characteristics and study outcomes were compared between the NAC and control groups using a student *t* test for normally distributed quantitative variables, a Mann–Whitney test for non-normally distributed quantitative variables, and a Chi-square test (Fisher or Monte Carlo) for categorical data. The significance of change within the two studied groups was analyzed using a paired *t* test for normally distributed quantitative variables and Wilcoxon signed ranks test for non-normally distributed quantitative variables. All analyses were conducted using IBM SPSS software package version 20 (IBM Corp, Armonk, NY). A statistical significance level of *p* value < 0.05 was used to assess the significance of the results obtained.

## Results

### Study population

**Fig. 1:**
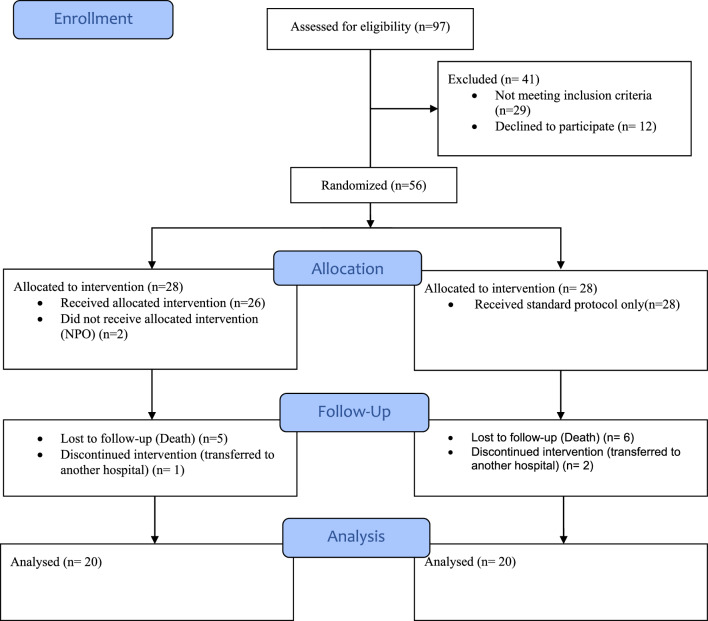
Flowchart of the trial

During the study, a total of 97 patients with moderate to severe TBI presenting to the critical care resuscitation unit at the Alexandria Main University Hospital were screened for eligibility. Forty-one patients were excluded, with 29 not meeting the inclusion criteria and 12 declining to participate. While 56 remaining patients were randomized and allocated into either NAC group or control group via block method randomization. In NAC group 2 patients were ineligible for (NAC) administration, 5 were lost to follow-up and only 1 discontinued the study. In the control group, 8 patients were lost to follow-up due to death or transfer to another hospital. Data from patients who were ineligible for NAC administration, lost to follow-up, or discontinued the study were excluded. Ultimately, data from 20 patients in each group were included in the final analysis. The flow chart is shown in Fig. 1.

### Baseline demographics and clinical characteristics

At baseline, no statistically significant differences were observed between the NAC and control groups regarding demographic variables (age and sex) or laboratory parameters, including creatinine, hemoglobin (HGB), White Blood Cells (WBCs), AST, ALT, and INR. In addition, the severity of TBI & the scoring of RTS, GCS, and Rotterdam showed no statistically significant difference between the two groups. (Table 1).

**Table 1:** Baseline demographics and clinical characteristics of NAC group and control group

	NAC group (*n* = 20)	Control group (*n* = 20)	*p*
*Sex*			
Male	17 (85%)	19 (95%)	0.605
Female	3 (15%)	1 (5%)	
Age (years)	36.15 ± 15.29	40.35 ± 19.60	0.455
*Trauma*			
RTA	18 (90%)	14 (70%)	0.235
FFH	2 (10%)	6 (30%)	
*Surgery*			
No	14 (70%)	16 (80%)	0.465
Yes	6 (30%)	4 (20%)	
GCS	6.50 (5.0 to 7.50)	8.0 (5.0 to 10.0)	0.127
Scr	0.85 (0.65 to 1.05)	0.85 (0.70 to 1.15)	0.495
HGB	12.58 ± 2.11	12.71 ± 2.08	0.845
WBCs	16.82 ± 6.71	15.65 ± 6.0	0.564
ALT	32.50 (27.0 to 47.50)	39.50 (23.50 to 57.0)	0.904
AST	58.50 (48.0 to 75.0)	54.50 (31.0 to 77.50)	0.478
INR	1.10 (1.02 to 1.23)	1.12 (1.0 to 1.41)	0.758
RTS	6 (5 to 6)	6 (5 to 7)	0.481
*Rotterdam score*			
1	6 (30%)	9 (45%)	0.834
2	7 (35%)	6 (30%)	
3	5 (25%)	3 (15%)	
4	2 (10%)	2 (10%)	
*Severity of TBI*			
Moderate	5 (25.0%)	9 (45.0%)	0.185
Severe	15 (75.0%)	11 (55.0%)	

Continuous variables were expressed as mean ± SD, while qualitative variables were expressed as numbers and percentages (No. and %)

*p p* value for comparing between NAC group and control group, *NAC* *N*-acetylcysteine, *RTA* road traffic accident, *FFH* fall from height, *GCS* Glasgow Coma Scale, *Scr* serum creatinine, *HGB* hemoglobin, *WBCs* white blood cells, *ALT* serum alanine aminotransferase, *AST* serum aspartate aminotransferase, *INR* international normalized ratio, *RTS* Revised Trauma Score, *TBI* traumatic brain injury

### Effect of NAC on IL-6, MDA, NSE and S100B

Inflammatory marker IL-6 was statistically non-significant at baseline between the NAC group and control group (*p* = 0.072). Although on day 7, NAC group demonstrated a significant reduction in both median values of IL-6 level (84.28 vs 87.83) & percentage change (− 16.78% vs − 2.56%) compared to control (*p* = 0.040 and *p* = 0.001, respectively). Moreover, on day 7, NAC group showed a significant reduction in IL-6 levels compared to their corresponding baseline values (*p* < 0.001), while no significance was noticed in the control group between baseline and day 7 (*p* = 0.247) (Table 2).

**Table 2:** Effect of NAC on IL-6, MDA, NSE, and S100B

	NAC group (*n* = 20)	Control group (*n* = 20)	*p*_*0*_
*IL-6*			
Baseline	103.4 (96.38 to 114.7)	92.86 (84.64 to 103.2)	0.072
Day7	84.28 (79.52 to 89.93)	87.83 (82.93 to 99.74)	0.040*
% Change	− 16.78(− 24.19 to − 8.44)	− 2.56 (− 11.69 to 3.03)	0.001*
*p*	*p* < 0.001*	*p* = 0.247	
*MDA*			
Baseline	7.11 ± 0.86	6.64 ± 1.07	0.136
Day7	5.55 ± 0.98	5.86 ± 1.13	0.363
% Change	− 21.81 ± 10.91	− 9.94 ± 21.69	0.049*
*p*	*p* < 0.001*	*p* = 0.030*	
*NSE*			
Baseline	16.90 (11.30 to 19.94)	11.43 (10.64 to 12.95)	0.060
Day7	7.61 (6.97 to 10.57)	10.30 (8.44 to 15.12)	0.049*
% Change	− 46.66 (− 61.79 to − 31.53)	− 14.68 (− 24.65 to − 4.34)	< 0.001*
*p*	*p* < 0.001*	*p* = 0.025*	
*S100B*			
Baseline	1.33 (0.91 to 1.85)	0.96 (0.78 to 1.28)	0.102
Day7	0.88 (0.70 to 1.28)	0.71 (0.64 to 1.17)	0.512
% Change	− 30.88(− 49.77 to − 0.95)	− 20.90(− 25.97 to − 8.09)	0.060
*p*	*p* = 0.003*	*p* = 0.204	

The normally distributed quantitative variable was expressed as mean ± SD. While non normally distributed quantitative variables were expressed as median (IQR). *p*_*0*_ *p* value for comparing between NAC group and control group, *p* *p* value for comparing between baseline and day 7 within the same group. %change: percentage change = [(values on day 7 − values at baseline)/values at baseline]*100

*NAC* *N*-acetylcysteine, *IL-6* Interleukin-6, *MDA* malondialdehyde, *NSE* neuron-specific enolase

*Statistically significant at *p* ≤ 0.05

Concerning oxidative stress marker, MDA, no significant differences were observed between the NAC and control groups at baseline or on day 7 (*p* = 0.136, 0.363, respectively). A significant reduction was demonstrated between the baseline and day 7 within both control group (*p* = 0.03) and NAC group (*p* < 0.001). Importantly, the percentage change in MDA levels was significantly greater in the NAC group compared to the control group on day 7, highlighting the more pronounced effect of NAC in reducing oxidative stress (− 21.81 vs 9.94, *p* = 0.049) (Table 2).

Both neuronal damage markers, NSE and S100B, showed no significant statistical difference between NAC group and control group at baseline (*p* = 0.060 and *p* = 0.102, respectively). However, on day 7, the median level of NSE in NAC group demonstrated a significant decrease compared to the control group (*p* = 0.049) and their corresponding baseline values (*p* < 0.001). Moreover, the NAC group showed a significant reduction in the percentage change of NSE levels compared to control group (− 46.66% vs − 14.68%, *p* < 0.001). On the other hand, although both values of S100B and percentage change revealed no statistically significant difference between NAC and control group on day 7 (*p* = 0.512 and *p* = 0.060 respectively) the significant reduction was shown in median values of S100B in NAC group on day 7 compared to its corresponding baseline (*p* = 0.003). In contrast, the control group showed no significant decrease in S100B levels between baseline and day 7 (*p* = 0.204) (Table 2).

### Effect of NAC on clinical outcomes

The patients in NAC and control groups showed a significant improvement in consciousness level on day 7 compared to baseline as GCS was elevated in both groups compared to corresponding values (*p* = 0.001 and *p* = 0.032, respectively). Despite no statistically significant difference in GCS scores was observed between the two groups on day 7 (*p* = 0.602), the percentage change in GCS levels was significantly higher in the NAC group compared to the control group on day 7 (*p* = 0.003) (Table 3).

**Table 3:** Effect of NAC on GCS

GCS	NAC group (*n* = 20)	Control group (*n* = 20)	*p*_*0*_
baseline	6.35 ± 1.76	7.65 ± 2.66	0.127
Day 7	9.85 ± 3.84	8.95 ± 4.38	0.602
*p*	*p* = 0.001*	*p* = 0.032*	
% Change	53.50 ± 49.37	12.55 ± 28.53	0.003*

Data were expressed by mean ± SD. *p*_*0*_ *p* value for comparing between NAC group and control group, *p* *p* value for comparing between baseline and day 7 in each group. %change: percentage change = [(values on day 7 − values at baseline)/values at baseline]*100

*NAC* *N*-acetylcysteine, *GCS* Glasgow Coma Scale

*Statistically significant at *p* ≤ 0.05

Regarding the effect of NAC on ventilation needs and duration, there was no statistically significant difference between the NAC and control groups concerning the need for mechanical ventilation, with 30% of patients in the NAC group and 25% in the control group not requiring ventilation (*p* = 0.723). Additionally, the duration of ventilation did not differ significantly between the two groups, with a median of 3.50 days (IQR 1.0–19.0) in the NAC group and 10 days (IQR 2.0–16.5) in the control group (*p* = 0.505). However, a significant difference was observed in the ICU length of stay, with the NAC group having a shorter stay (median = 14.5 days and IQR 9.0–24.50 days) compared to the control group (median 21 days and IQR 15.0–32.0 days, *p* = 0.038), suggesting that NAC may contribute to a reduced ICU stay (Table 4).

**Table 4:** Effect of NAC on clinical outcomes

	NAC group (*n* = 20)	Control group (*n* = 20)	*p*
Ventilation needs	No. (%)	No. (%)	
No	6 (30%)	5 (25%)	
Yes	14 (70%)	15 (75%)	0.723
Ventilation days			
Median (IQR)	3.50 (1.0–19.0)	10.0 (2.0–16.50)	0.505
ICU length of stay			
Median (IQR)	14.50 (9.0–24.50)	21.0 (15.0–32.0)	0.038*

Data were expressed by median

*IQR* inter quartile range, *p p* value for comparing between the two studied groups, *NAC* *N*-acetylcysteine, *ICU* intensive care unit

*Statistically significant at *p* ≤ 0.05

**Fig. 2:**
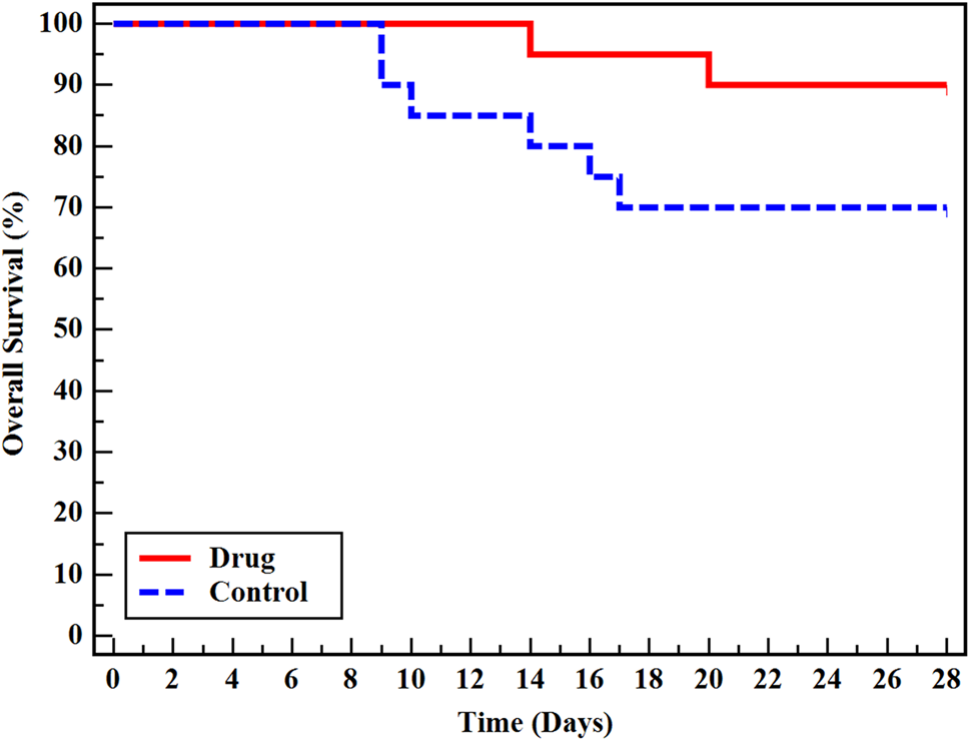
Kaplan–Meier survival curve for 28-days survival in NAC group and control group (*p* = 0.103)

With respect to survival analysis, the Kaplan–Meier curve revealed that there was no statistically significant difference between NAC and control group within 28 days of follow-up (*p* = 0.103). However, the NAC group demonstrated higher overall survival rates relative to the control group 90% vs 70% on day 28. This may suggest a potential survival benefit associated with NAC treatment (Fig. 2).

### Adverse events and monitoring

All possible adverse events were monitored clinically by the healthcare team and by lab investigations of the two studied groups during the study period. All adverse events presented during the trial are commonly present in ICU patients. Including, chest infection, wound infection, hematemesis, electrolytes disturbances, convulsions, and acute kidney injury (AKI) are presented in both groups. There was no statistically significant difference in adverse events between the two studied groups (Table 5).

**Table 5:** Comparison between the two studied groups according to adverse events

Adverse events	NAC group (*n* = 20)	Control group (*n* = 20)	*p*
	No. (%)	No. (%)	
HAP	1 (5)	4 (20)	0.342
VAP	2 (10)	3 (15)	1.000
Wound infection	2 (10)	1 (5)	1.000
AKI	1 (5)	4 (20)	0.342
Coagulopathy	1 (5)	2 (10)	1.000
Convulsions	4 (20)	5 (25)	1.000
Mild hematemesis	2 (10)	2 (10)	1.000
UTI	1 (5)	1 (5)	1.000
Hypernatremia	2 (10)	3 (15)	1.000
Hyperbilirubinemia	1 (5)	2 (10)	1.000
Hypokalemia	4 (20)	2 (10)	0.661
Hypocalcemia	0 (0)	3 (15)	0.231

Data were expressed as numbers and percentages (no. and %), *p p *value for comparing between the two studied groups

*NAC* *N*-acetylcysteine, *HAP* Hospital-acquired pneumonia, *VAP* ventilator-acquired pneumonia *AKI* acute kidney injury, *UTI* urinary tract infection

*Statistically significant at *p* ≤ 0.05

## Discussion

Despite all advancements in diagnostic and surgical techniques, there is currently no definitive available treatment for TBI, and the only main therapeutic goal is symptomatic treatment, prevention of secondary injury, and control progression of TBI complications (Lozano et al. 2015). Targeting neuroinflammation during the secondary wave of biochemical pathways in secondary injury represents a critical window for intervention. A variety of anti-inflammatory and neuroprotective drugs have been extensively studied and reported in both animal models and TBI patients, spanning preclinical and clinical levels of research (Kalra et al. 2022). Numerous experimental studies have revealed that NAC is a promising choice in ameliorating complications of TBI as it has anti-inflammatory, neuroprotective and antioxidant mechanisms (Bavarsad Shahripour et al. 2014).

Following TBI, neuronal damage creates an inflammatory microenvironment, triggering the release of damage-associated molecular patterns and cytokines. These signals recruit both local microglia and circulating macrophages. IL-6, a crucial cytokine, is released by microglia, astrocytes, and neurons after TBI. While it is undetectable in the brain under normal physiological conditions, its acute release in response to injury is well-documented (Ooi et al. 2022). Elevated IL-6 levels are associated with poor prognosis and worse outcomes in TBI patients (Ooi et al. 2022). In our study, NAC significantly reduced IL-6 levels from baseline compared to control group which may be translated into less inflammation-induced damage and better short-term outcomes. These results conform with results obtained by (Sabetghadam et al. 2020) showing that NAC can reduce IL-6 compared to control groups in patient neuronal damage due to stroke.

Since there is a positive association between MDA and mortality of patients with TBI, and thus, reducing oxidative stress using NAC may positively affect patient outcomes (Lorente et al. 2015). Our study showed that NAC can significantly reduce oxidative stress following TBI compared to control group which may improve prognosis. These results conformed with results obtained by Zhang et al. (2018) where NAC improved oxidative stress and inflammatory response leading to a decrease in MDA levels in NAC group compared to control group in patients with CAP (Zhang et al. 2018). Despite the reduction of MDA levels showed no significant difference between the two groups in 28-day mortality, this may be due to the small sample size and the inability of long-term outcomes monitoring.

NSE is a glycolytic enzyme primarily found in neurons and other ectodermal cells. It is considered one of the most promising biomarkers for assessing brain damage and monitoring recovery following TBI (Isgrò et al. 2015). NSE levels can be measured in both cerebrospinal fluid and peripheral blood samples. Numerous studies have shown an inverse correlation between serum NSE levels and GCS score. Our study showed a significant decrease of serum NSE from baseline in NAC group compared to control group and also an increase in GCS in NAC group. In line with our study, data provided by (Sabetghadam et al. 2020), showed a significant decrease in NSE in NAC treatment group in stroke patients using Hoffer et al. regimen.

S100B is primarily expressed in astrocytes so it is released extracellularly following astroglial injury due to both primary and secondary TBI (Thelin et al. 2017). Elevated serum S100B levels were correlated to worse clinical outcomes and GCS (Da Rocha et al. 2006). Reducing S100B levels following TBI has been shown to reduce neuroinflammation, cell loss, and neurologic dysfunction in the TBI model (Kabadi et al. 2015). Thus, reducing S100B may improve the long-term disability and decrease complications of TBI and may be a useful therapeutic target. In our study, there was a significant decrease in serum S100B levels in NAC group in contrast to control group suggesting an improved clinical outcome with NAC. NAC may have reduced cell damage through antioxidant properties and thus reduced S100B release (Leclerc et al. 2007).

GCS is a scoring system for neurological assessment of the extent of impaired consciousness in all types of acute medical and trauma patients (Basak et al. 2023). NAC caused a significant increase in GCS in favor of NAC group compared to control group (*p* = 0.001 and *p* = 0.032 respectively) indicating a positive effect of NAC on short-term clinical outcomes of TBI.

This is the first prospective randomized controlled study to investigate the effect of NAC in moderate to severe TBI patients. Our results are consistent with both previous reports and the expected pharmacological action of NAC. NAC can through its anti-oxidant and anti-inflammatory action reduce markers of neurological, oxidative, and inflammatory damage compared to the control group with significantly better clinical improvement as demonstrated by GCS over the course of 7 days. Our study findings showed a positive effect of the drug in reducing the measured biomarkers and improvement of GCS and ICU length of stay. This study provides important insights into the increasing role of antioxidants in TBI patients. As a result, to reduce TBI resulting morbidity it is suggested that the use of antioxidants as NAC be considered in all patients.

There are some previous interventional studies evaluating the role of NAC as anti-inflammatory, antioxidant, and neuroprotective in different populations. Hoffer et al. designed a double-blind placebo-controlled human trial to evaluate the efficacy of NAC in patients with blast-induced mild TBI. The primary outcome was the remission of mTBI sequelae symptoms: dizziness, hearing loss, headache, memory loss, sleep problems, and neurocognitive impairment seven days following the explosion (Hoffer et al. 2013). NAC was significantly better than placebo with an 86% likelihood of symptom remission with no reported adverse effects (Hoffer et al. 2013). Another study that used a combination of NAC, multivitamins, and weight loss on retired professional football players showed significant improvements compared to baseline in cognitive function, processing speed and accuracy, attention, and memory (Amen et al. 2011). Similar results were obtained by Sabetghadam et al. (2020), where NAC use in patients with acute stroke for four days using the Hoffer et al. regimen was associated with a significantly favorable functional outcome compared to placebo 90 days after stroke. NAC treatment significantly decreased serum levels of inflammatory biomarkers such as IL-6, MDA, and NSE (Sabetghadam et al. 2020).

This study has several limitations, including limited sample size, the single-center nature of the study, an open-label design, using of oral regimens instead of the IV form of the drug which may yield better results due to better bioavailability.

## Conclusion

This study showed that NAC effectively reduced inflammatory marker IL-6, oxidative stress marker MDA, and markers of neurological damage NSE and S100B, and also improved clinical outcomes such as GCS score and ICU length of stay. Their reduction may be translated into the neuroprotective effect of NAC which helps in improving morbidity and mortality, also it is related to the anti-inflammatory and antioxidant effect of NAC. It also showed better clinical improvement of GCS and decreased ICU length of stay. In conclusion, NAC can be considered a supportive treatment for moderate to severe TBI due to its anti-inflammatory, antioxidant, and neuroprotective effects that may improve clinical outcomes in such a special population of TBI patients.

## Data Availability

Data will be made available on request.
